# Using Quality Improvement and Workflow Analysis to Successfully Implement Evidence‐Based Interventions to Increase Colorectal Cancer Screening Rates

**DOI:** 10.1002/cam4.71634

**Published:** 2026-02-13

**Authors:** Mark M. Macauda, Lisa A. Scott, Rebecca P. Eaddy, Ljubitca S. Fadic Quijano, Tracie R. Lewis, Nazratun N. Monalisa, Annie Thibault

**Affiliations:** ^1^ Colorectal Cancer Prevention Network at the University of South Carolina Columbia South Carolina USA; ^2^ Center for Applied Research and Evaluation at the University of South Carolina Columbia South Carolina USA

**Keywords:** colorectal cancer screening, evidence‐based interventions, quality improvement, workflow analysis

## Abstract

**Background:**

Colorectal cancer (CRC) is the third leading cause of cancer deaths in the United States for men and women combined but is preventable with timely screening. Evidence‐based interventions (EBIs) provide promising opportunities to increase screening. There are few descriptive examples of the processes used to assess and implement EBIs to increase CRC screening.

**Project Description:**

The Colorectal Cancer Prevention Network (CCPN) in South Carolina facilitated an intensive quality improvement technical assistance project aimed to increase CRC screening in 25 primary care clinics. In this paper we provide a detailed description of the process used to implement EBIs, report on the changes in CRC screening rates, and examine the impact of the interventions across clinics with different attributes (such as clinic size and rurality).

**Methods:**

We used Chi‐square to explore changes in screening rates from baseline to years two and three of clinic implementation. We used Difference‐in‐Differences analysis to assess changes in screening rates from baseline to third year for clinics with different attributes.

**Results and Conclusions:**

Across all clinics, the CRC screening increased from 45% to 51% (*p* < 0.05) from baseline to third year of participation. Sixteen of out 25 clinics saw an increase in screening rates for their second year, and 14 out of 25 saw an increase in their third year. Clinics with smaller patient populations, rural clinics, clinics with fewer uninsured patients, and clinics with lower baseline rates saw greater percentage point improvements. Clinics onboarded in the second year saw the lowest gains. We conclude that a structured tailored approach to the selection of EBIs can have positive effects on CRC screening rates, but positive change may vary depending on clinic attributes.

## 1 Introduction

Colorectal cancer (CRC) is the second leading cause of cancer deaths in the United States and in South Carolina [1, 2, 3, 4] for men and women combined. Across the United States, the incidence rate (per 100,000) of CRC between 2017 and 2021 was 36.4, and mortality was 12.9 [5]. In South Carolina, our project location, CRC incidence from 2017 to 2021 (35.6) was slightly lower than the national rate, but mortality (13.7) was higher [5]. CRC is highly preventable and treatable when individuals comply with timely screening [6, 7]. Unfortunately, CRC screening participation remains low across the United States [8, 9, 10, 11] and research suggests that complex sociodemographic, psychosocial, and economic barriers to screening can be difficult to mitigate [12, 13, 14, 15, 16, 17].

Since the National Colorectal Cancer Roundtable launched its national campaign to increase CRC screening to “80% in every community” [18], the emergence of new interventions and screening modalities has offered promising opportunities to increase CRC screening compliance [11, 19]. Evidence‐based interventions like provider assessment and feedback, provider reminders, patient reminders, and reducing structural barriers to care [9, 20] have been associated with increases in CRC screening rates [5, 9]. However, evidence‐based interventions remain underused in primary care clinics.

The Centers for Disease Control and Prevention (CDC) Colorectal Cancer Control Program (CRCCP) funded a state‐level program to increase CRC screening participation by supporting primary care clinic implementation and usage of evidence‐based interventions (EBIs) to address patient‐specific barriers to CRC screening. The project “SC Communities Unite to Increase CRC Screening” (SC Communities Unite) was carried out by the Colorectal Cancer Prevention Network (CCPN) at the University of South Carolina. The CCPN provided technical assistance (TA) to primary care clinics in selecting, implementing, and refining evidence‐based interventions using robust quality improvement processes.

While there are several examples in the literature of the implementation of EBIs to increase colorectal cancer screening rates [21, 22, 23, 24, 25, 26, 27], as well as the implementation of evidence‐based practice more broadly [28, 29], there are few descriptive examples of the specific *processes* used to integrate EBIs into clinic workflow for CRC screening. In this paper, we (1) describe, in detail, the quality improvement process used by the CCPN to integrate EBIs into the workflow of partner clinics. (2) assess the impact of our process on clinic CRC screening rates (3) explore differences in screening rate changes based on clinic attributes (such as clinic size, location, number of uninsured patients, and perceived readiness).

## 2 Materials and Methods

### 2.1 Clinic Enrollment

Clinics included 23 from four Federally Qualified Health Center (FQHC) systems, and two from one hospital system. Clinic enrollment in the project was staggered over a 3‐year period, starting on June 30th of each project year. “Cohort 1” was comprised of 13 clinics onboarded in 2020, “Cohort 2” comprised eight clinics onboarded in 2021, and “Cohort 3” comprised four clinics onboarded in 2022. All health systems had clinics onboard in Cohort 1.

### 2.2 Outcome Assessment Data

To assess FQHC clinic CRC screening rates, we utilized the CRC Screening Uniform Data Set (UDS) [30] quality measures collected through electronic medical records. Screening rates were further validated by each FQHC clinic prior to analysis. For hospital system primary care clinics, we used data collected from the Healthcare Effectiveness Data and Information Set (HEDIS) [31] reporting, which was also validated by the clinics. Both sources of data reported CRC screening rates for an entire calendar year (Jan–Dec) and were used as our official CRC screening rate for CDC project reporting. The Cohort 2 and Cohort 3 clinic CRC rates for 2023 included patients ages 45–74 in response to the change in screening guidelines recommended by the United States Preventive Services Taskforce (USPSTF) in 2021 [9] and present in our UDS and HEDIS measures for 2023. Thus Cohort 2 included 45–49 year olds in their third participation year, and Cohort 3 included them in their second participation year.

We used the following data points from UDS and HEDIS in our assessment of screening outcome: (1) trailing year baseline screening rate collected the year prior to each clinic's project onboarding; (2) trailing year screening rate from each clinic's second year of participation after implementation of interventions; and (3) trailing year screening rate from the third year of clinic's participation, after 2 years of intensive monthly technical assistance sessions and transition to quarterly meetings.

In addition to assessing the clinic, system, and overall CRC screening outcomes, we wanted to assess whether some clinic attributes correlated with variation in clinic outcomes. These included the number of screening‐eligible patients averaged across project years as a proxy measure of clinic size [32]; (2) whether the clinic was situated in an urban or rural setting [33], (3) the number of uninsured screening‐eligible patients averaged across project years [34], (4) whether the clinic belonged to Cohort 1, Cohort 2 or Cohort 3, since the second two cohorts included 45–49 year olds in their screening rates during their first 3 years [9], (5) the average score from pre‐implementation clinic readiness assessments [35] and (6) CRC screening rate at baseline (to examine whether the initial screening rate affected screening rate increases).

For CRC screening rates for continuous clinic characteristics, such as number of patients seen, baseline screening rate, readiness score, and number of uninsured patients, we calculated the median value for each attribute and split the clinics into two groups, above and below the median. The median value was assigned to the lower group. For categorical variables, such as rurality (self‐reported by clinics during onboarding) and cohort, the clinics were divided into the respective groups.

Readiness assessments (which were part of clinic onboarding, see below) included questions that were formatted on a 4‐point scale from 1 = Disagree to 4 = Strongly Agree (Do not know = 0 was removed prior to analysis). Thus, the higher the scores, the greater the degree of perceived readiness for intervention implementation. The maximum score was 80. There were 20 questions including “Promoting CRC screening is a priority for my clinic”, “My clinic monitors colorectal cancer screening rates monthly,” and “This clinic's staff and providers are receptive to making practice changes to match the system's priorities”. Readiness assessments were completed yearly by staff from each clinic. All clinic staff were invited to complete the assessments anonymously. For our analysis we averaged the readiness assessment responses across the first 3 years for each clinic and summed the total score from the average. This helped to address missing data and to gather as many staff datapoints as possible.

### 2.3 Statistical Approach

To test for statistical significance in CRC screening rate changes between baseline and subsequent years, we used Microsoft Excel to calculate Chi‐square contingency tables. Significance was set at a threshold of *p* < 0.05. We used the total number of screened patients and the total number of unscreened patients for each category of interest (Individual clinics, systems, overall screening rate) from baseline compared to years two and three. To assess the differential performance of clinics, we used general linear modeling in SPSS 29 to create a Difference‐in‐Differences measure. We compared the number of screened patients (numerator) vs. eligible patients (denominator) across the two time periods of baseline and the third year of participation for each of the clinic groupings by attribute (e.g., low vs. high readiness score, low vs. high number of uninsured patients, rural vs. urban). Significance was tested using Wald Chi‐square. Significance was set at a threshold of *p* < 0.05. We chose to compare baseline to the third year since it reflected the clinic's ability to sustain CRC rates after the conclusion of intensive technical assistance. For all variables we used the validated clinic data as it was reported to CDC.

## 3 Description of SC Communities Unite EBI Implementation Process

From 2020 to 2025, CCPN worked with the staff and leadership of our four FQHCs and one hospital system to foster a supportive and cohesive approach in the selection, implementation, and quality improvement of evidence‐based interventions in 25 primary care clinics. Clinics were chosen in consultation with the health systems. CRC screening rates were below 60% at project baseline for all 25 clinics. All clinics participated in four project phases of quality improvement (QI) activities that were tailored to each clinic based on their location, size, patient population profile, staffing capacity, and baseline CRC screening rate.

To gather data to support the continuous quality improvement process, FQHC clinics used the cloud‐based health information technology platform Azara [36] to extract electronic medical record data, whereas the hospital system used their electronic medical record system with a built‐in population health dashboard. In partnership with the South Carolina Primary Healthcare Association, the CCPN developed a monthly customized Azara Data Reporting and Visualization System (DRVS) report that summarized trailing year aggregate CRC screening rates by patient demographics, insurance status, and social determinants of health. These data extractions provided clinics with very precise CRC screening data and were used to guide decisions around intervention enhancement. Lucidchart [37], a cloud‐based visual collaborative platform, was used to document processes, guide interactive technical assistance sessions, and track outcome measures. Lucidchart provided our TA team and clinic staff with the ability to create and edit virtual interactive diagrams, offering a visual component used by the clinics to discuss their quality improvement steps.

### 3.1 Phase 1: Identify Areas for Improvement

This phase fostered a comprehensive clinic‐level review of each step of their CRC screening process at project baseline. First, clinic staff and leadership completed readiness and capacity assessments to gather specific clinic‐level information (e.g., number of staff by role/position, type of CRC screening modalities ordered, use of EMR and health information technology tools to monitor CRC screening rates, consistency of patient CRC risk assessment, clinic CRC screening protocols, staff knowledge and preparedness to implement interventions, and perceptions of CRC screening prioritization by staff and leadership). Readiness and Capacity assessments were also completed by each clinic yearly. Next, clinic leadership and staff attended a virtual workshop conducted by the American Cancer Society (QI bootcamp) where participants were provided an overview of the Institute for Healthcare Improvement's (IHI) Model for Improvement [38]. Additionally, participants learned key team building strategies, goal setting, and the role of data monitoring in clinical quality improvement.

**FIGURE 1:**
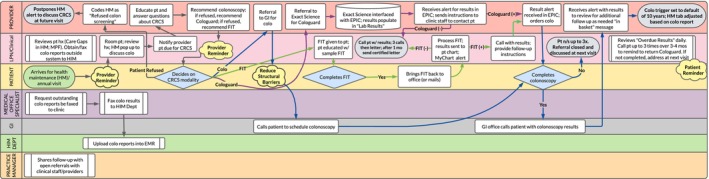
Swim lane workflow diagram.

Following QI bootcamp, each clinic began monthly technical assistance sessions. The first activity required clinic staff to write an aim statement, which was the foundation of each individual clinic's desired CRC screening outcomes. Next, clinics joined a process mapping session in Lucidchart to capture their current CRC screening workflow. Clinic staff delineated clinical roles (e.g., provider, medical assistant, nurse, lab, reception, and support staff) and responsibilities (e.g., completion of patients' risk assessment, screening order creation, reception and upload of screening results, and delivery of results to patient) in the CRC screening process. Subsequently, the CCPN team converted the documented workflow into a current state “swim lane” workflow diagram (Figure 1). This diagram allowed clinic staff to assess potential process gaps and barriers to effective CRC screening.

**FIGURE 2:**
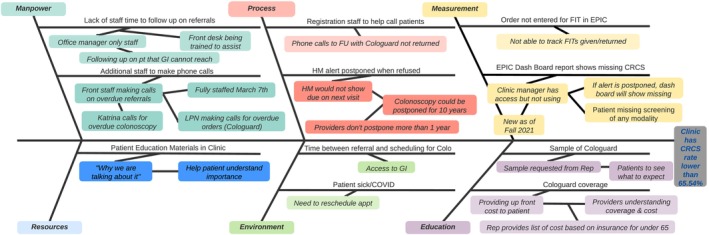
Fishbone diagram.

Next, clinics used the defined workflow to identify screening challenges using a root cause analysis. The TA team facilitated clinic staff through a series of questions to identify barriers that impacted CRC screening completion rates based on specific categories (e.g., staffing, process, measurement, education, environment, and resources) using a “fishbone diagram” exercise (See Figure 2). Once identified and categorized, clinics were asked to prioritize the screening barriers to address.

Using the results obtained from these activities, clinics selected at least two of four evidence‐based interventions, which were incorporated into their clinic's practice workflow. In Lucidchart, clinics recorded which interventions they selected, how the interventions addressed screening barriers, and detailed how each intervention would be implemented. Of the 25 clinics, 21 selected patient reminder, 22 selected provider reminder, 14 selected provider assessment and feedback, and seven selected reducing structural barriers. The median number of interventions implemented by clinics was three.

### 3.2 Phase II: Implementation

Once clinics selected their interventions and began implementation, clinic staff were asked to complete Plan‐Do‐Study‐Act (PDSA) cycles to identify potential areas for process improvement and make small process adaptations that could improve their interventions [38]. Clinics were encouraged to use monthly CRC screening data reports to monitor CRC screening outcomes. After PDSA cycles were conducted, clinic staff met with the TA team to discuss and evaluate the impact that process adjustments had on screening outcomes and decide if further adjustments were warranted.

### 3.3 Phase III: Sustainability

The sustainability phase ensured clinics' intervention implementation and clinical workflow could be sustained beyond the scope of the project. With the support of the CCPN TA team, clinics were asked to review their initial swim lane workflow diagram to document where it was refined to achieve optimal intervention implementation. The TA team created a future state process map that could be used as a reference and to train new clinic staff, ensuring consistency and sustainability of intervention implementations over time.

As clinics transitioned to the sustainability phase, they created a sustainability plan with the support of the Washington University in St. Louis Center for Public Health Systems Science (WashU). Using WashU's Clinical Sustainability Assessment Tool (CSAT) [39], clinics assessed their capacity to sustain interventions based on seven domains known to impact organizational sustainability (Engaged Staff & Leadership, Engaged Stakeholders, Organizational Readiness, Workflow Integration, Implementation & Training, Monitoring & Evaluation, and Outcomes & Effectiveness). Sustainability plans were reviewed by clinic staff after 6 months. Twelve months later, clinics completed a second CSAT to further evaluate and monitor the sustainability of interventions within the clinic workflow.

### 3.4 Phase IV: Medical Neighborhood

This final phase of the project turned staff attention outside of their individual clinics to determine if untapped external medical and public health entities and/or resources were available and could further support screening intervention implementation and increases in CRC screening completion (access to follow‐up colonoscopy, community transportation services, etc.). In support of this phase, CCPN's TA team assisted clinics in completing a local environmental scan and stakeholder mapping exercise in Lucidchart. The goal was to identify and prioritize community partnerships that played a role in their patients' completion of CRC screening. Clinics assigned stakeholders (such as gastroenterology practices, community organizations, pharmaceutical organizations, and other healthcare organizations) into quadrants by level of interest (low‐high) and power to enact change (low‐high). The systems could then use this information to guide sustainability post‐project.

## 4 Screening Results

**FIGURE 3:**
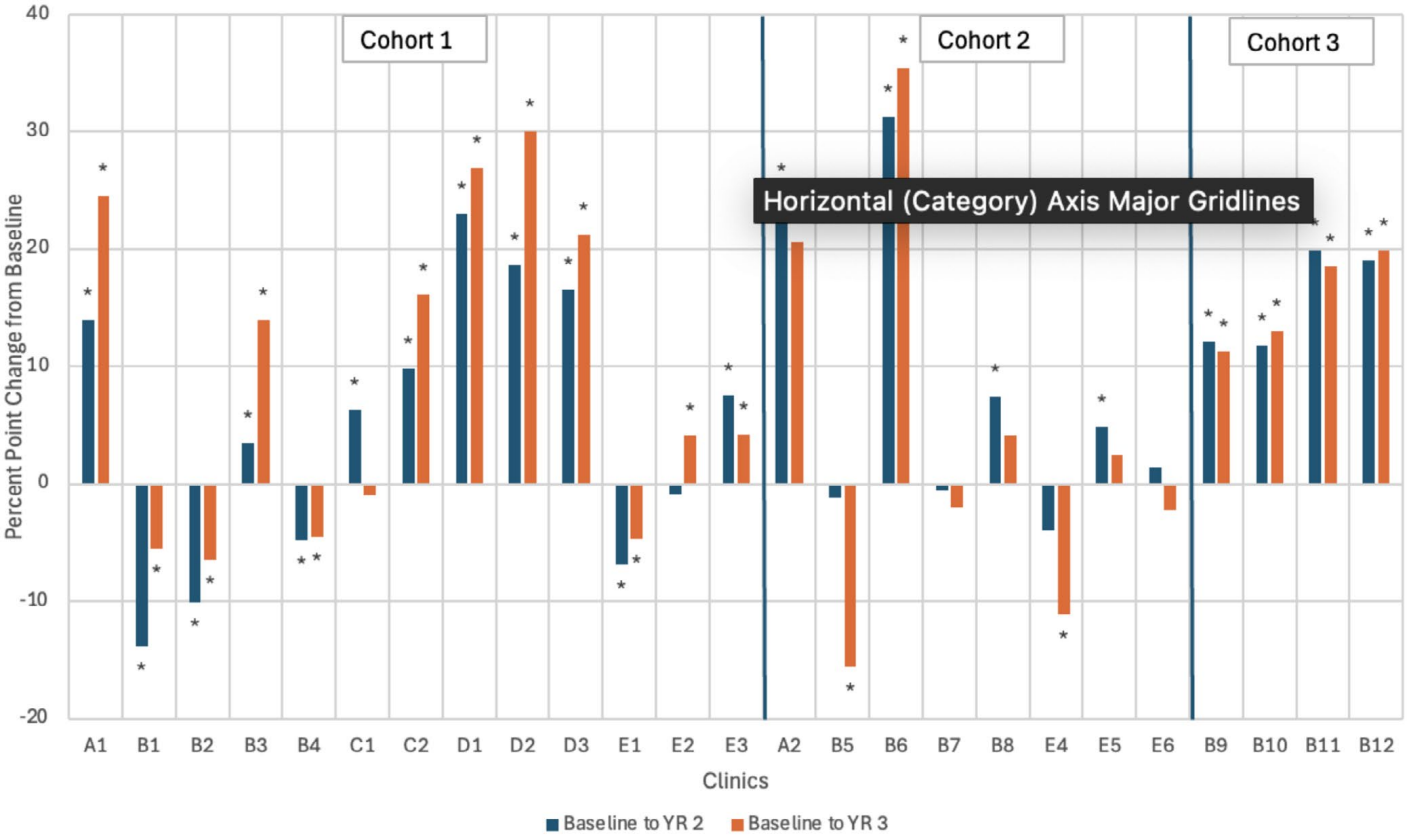
YR2 and YR3 CRC screening rate change from baseline (By Cohort).

Figure 3 shows each clinic's CRC rate changes from their individual baselines to their second and third years of participation. Of the 25 clinics, 16 clinics saw statistically significant increases in CRC screening rate from baseline to their second year of project participation, with a median positive change of 13.04 percentage points (SD = 7.91). Conversely, four clinics experienced a decrease in their CRC screening rate between baseline and their second year of participation, with a median decrease of 8.5 percentage points (SD = 3.95). Finally, five clinics did not see a statistically significant change in CRC screening rate between baseline and their second year of participation. In clinics' respective third year of participation, 14 clinics saw a significant positive change in their CRC screening rate from baseline, with a median increase of 19.22 percentage points (SD = 9.03). Conversely, six clinics saw their CRC screening rate decrease from baseline, with a median decrease of six percentage points (SD = 4.43). Five clinics saw no significant change.

Across all clinics, the CRC screening rate saw a positive statistically significant increase from 45% to 51%. Rate increases did vary significantly by health system, from a less‐than‐one percentage point increase to a 23‐percentage point increase. Four out of five systems saw a statistically significant increase in CRC screening rates from their baseline to their third year of participation. See Table 1, below.

**TABLE 1:** Rate changes by system.

System	Baseline CRC screening rate	YR‐2 CRC screening rate	YR‐3 CRC screening rate	% Point change baseline to YR‐3	Chi‐square	*p*
**A (2 Clinics)**	**46%**	**65%**	**69%**	**23**	**264.21**	**0.00**
**B (12 Clinics)**	**45%**	**50%**	**52%**	**7**	**90.59**	**0.00**
**C (2 Clinics)**	**55%**	**62%**	**57%**	**2**	**4.22**	**0.04**
**D (3 Clinics)**	**29%**	**38%**	**48%**	**19**	**162.55**	**0.00**
E (6 Clinics)	45%	45%	45%	< 1	0.94	0.33
**All patients**	**45%**	**50%**	**51%**	**6**	**213.74**	**0.00**

*Note:* Bold indicates statistical significance at *p* < 0.05.

The clinic characteristics that we tested showed that all were significant except the readiness score. Clinics with larger patient populations had a higher baseline rate (47% vs. 38%) but saw a smaller baseline to third year change (five percentage points vs. 12). Similarly, clinics that started at a lower baseline saw an overall lower third year rate than clinics that started at a higher baseline, but saw a greater baseline to third year rate increase (11 percentage points vs. four). Compared to Cohort 1, Cohort 2 clinics started at a slightly lower baseline and saw a smaller percentage point gain (three vs. 10). Clinics in Cohort 3 started at a lower baseline (31% vs. 48%) but saw a greater percentage point gain (14 vs. seven points). Similarly, rural clinics started at a lower baseline (41% vs. 48%) than urban clinics but saw a greater percentage point increase (11 vs. three percentage points). Finally, clinics with a greater percentage of uninsured patients had a slightly higher initial screening rate, but had smaller gains than those with fewer uninsured patients (four vs. nine points), see Table 2, below.

**TABLE 2:** Results of difference in differences analysis for clinic attributes.

Variable pairs	Baseline num.	Baseline denom.	Baseline rate	Yr3 num.	Yr‐3 denom.	Yr‐3 rate	Baseline to Yr‐3 change	Wald Chi‐square	Sig
Larger patient population	9807	20,784	47%	11,975	23,037	52%	5	69.908	< 0.001
Smaller patient population	3206	8433	38%	4605	9186	50%	12		
Lower readiness	7012	16,353	43%	9017	18,141	50%	7	0.057	0.812
Higher readiness	6001	12,864	47%	7563	14,082	54%	7		
Lower baseline	3982	12,043	33%	5723	12,949	44%	11	90.97	< 0.001
Higher baseline	9031	17,174	53%	10,857	19,274	56%	4		
Cohort1	9395	19,654	48%	11,534	21,199	54%	7	Reference	Ref
Cohort2	2391	5651	42%	3419	7473	46%	3	vs. Cohort1 19.439	0.002
Cohort3	1227	3912	31%	1627	3551	46%	14	vs. Cohort1 145.279	< 0.001
Rural	6138	14,926	41%	8875	17,140	52%	11	91.581	< 0.001
Urban	6875	14,291	48%	7705	15,083	51%	3		
Higher uninsured	5852	12,727	46%	7003	13,953	50%	4	34.70	< 0.001
Lower uninsured	7161	16,490	43%	9577	18,270	52%	9		

## 5 Discussion

Clinics enrolled in *SC Communities Unite* participated in intensive multi‐year quality improvement activities to select, implement, and enhance evidence‐based interventions to improve their clinic's overall CRC screening rates. Many clinics saw statistically significant increases in their CRC screening rates and maintained these increases across project years, and four out of the five systems saw statistically significant increases, including the system with the largest number of clinics. However, the system that did not see increases was our second largest with six clinics. Of these six, only two had positive significant changes from baseline to year three. While we did not have a control group of clinics for this project, it is worth noting that in FQHCs across South Carolina, the CRC screening rate was 44.08% in 2021 and 46.06% in 2024 [40], a gain of 1.98 percentage points, whereas for our participating clinics, the CRC screening rate went from 47.07% to 53.29%, a gain of 5.72 percentage points during the same period (these numbers are slightly different than our results above due to reporting by calendar year, when our clinics were in different implementation stages).

Our Difference in Differences analysis shows that clinic characteristics also correlate with performance. Those with smaller patient populations started with a lower baseline but saw greater gains, as did rural clinics (though these two factors are likely related), and clinics with fewer uninsured patients. The performance of the three cohorts also differed. Cohort 2 saw the smallest improvement to third year, and this may be because this cohort had to adjust to new screening guidelines that included 45–49 year olds. This change would have been part of Cohort 3's second year measures. We note that the readiness assessment was not predictive of clinic performance. This may be because the readiness assessment did not collect information that truly assessed readiness for this specific implementation, especially considering the degree to which the project, by design, increased implementation capacity through QI. Alternately, it could be because the scores were generally high and there was little variation, as the median score was 69 and the range was 60 to 76 out of a possible score of 80. This may be because clinic staff consistently felt ready to participate. Alternately, this may indicate a response bias, as clinic staff may have been reluctant to give a negative view of their respective clinics.

An interesting trend was that across different comparators, those that started out with a lower baseline CRC screening rate tended to see greater percentage point gains than those that started higher baselines (with the notable exception of readiness, which was not predictive, and membership in cohort 2, which is likely due to the inclusion of younger patients in screening). While such a pattern could reflect regression to the mean, the screening rate data used in our analysis represent trailing year data and thousands of patients, which reduces the likelihood of measurement errors or the chance that a random extreme measure is solely responsible for the observed pattern.

## 6 Limitations

Limitations of our evaluation study include that the clinic sample size was too small to explore the relationship between clinic attributes and outcome in statistical models, limiting us to univariate analysis. In addition, three clinics did not have baseline CRC rates available, so we used rates from those clinics' first year of participation, which were likely higher than baseline. Additionally, since our project was an intervention implementation, we did not have a comparison group. In addition, since this was an intervention implementation, we selected clinics based on need and consultation with health systems. We realize the implication that our results may not be generalizable to FQHC clinics more broadly. There also may be other factors that affected screening rates that are unreported in this evaluation.

## 7 Conclusions

Integrating effective evidence‐based interventions into a primary care clinic workflow to achieve increased CRC rates is time‐intensive and can be challenging when faced with a clinic environment where staff shortages, staff turnover, and leadership changes are common (a fact that became evident during the course of the project). A structured, tailored approach to clinical quality improvement processes where clinic staff are guided step by step through workflow assessment, identification of screening barriers, and data‐informed selection of EBIs can yield improved CRC screening rates despite obstacles faced by clinics. Though we believe that this work represents necessary initial steps to understand effective EBI implementation, additional work is necessary to understand how different attributes of clinics and patient populations influence successful implementation, and ultimately how to best support different types of clinics and patients in achieving desired CRC screening rates.

## Author Contributions

**Mark M. Macauda:** conceptualization (lead), formal analysis (lead), methodology (lead), writing – original draft (lead), writing – review and editing (equal). **Lisa A. Scott:** conceptualization (supporting), data curation (equal), project administration (equal), writing – review and editing (equal). **Rebecca P. Eaddy:** project administration (equal), writing – review and editing (equal). **Ljubitca S. Fadic Quijano:** project administration (equal), writing – review and editing (equal). **Tracie R. Lewis:** funding acquisition (lead), project administration (equal), writing – review and editing (equal). **Nazratun N. Monalisa:** data curation (equal), methodology (supporting), writing – review and editing (equal). **Annie Thibault:** funding acquisition (lead), project administration (equal), writing – review and editing (equal).

## Funding

This article was supported by Cooperative Agreement Number CRCCP‐RFA‐DP‐20‐2002 from the Centers for Disease Control and Prevention.

## Ethics Statement

This study was reviewed by the Institutional Review Board at the University of South Carolina and was deemed “not human subjects”. Review number Pro00145016.

## Conflicts of Interest

The authors declare no conflicts of interest.

## Data Availability

The data that support the findings of this study are available from the corresponding author upon reasonable request.

## References

[1] R. L. Siegel , A. N. Giaquinto , and A. Jemal , “Cancer Statistics, 2024,” CA: A Cancer Journal for Clinicians 74, no. 1 (2024): 12–49, 10.3322/caac.21820. 38230766

[2] National Cancer, Institute , “SEER*Explorer Application,” accessed October 15, 2024, https://seer.cancer.gov/statistics‐network/explorer/application.html?site=1&data_type=1&graph_type=2&compareBy=sex&chk_sex_3=3&chk_sex_2=2&rate_type=2&race=1&age_range=1&hdn_stage=101&advopt_precision=1&advopt_show_ci=on&hdn_view=0&advopt_show_apc=on&advopt_display=2#resultsRegion0.

[3] Office of Disease Prevention and Health Promotion , “Objectives and Data—Healthy People 2030|health.gov,” (n.d.), accessed October 15, 2024, https://health.gov/healthypeople/objectives‐and‐data.

[4] National Center for Health Statistics , “Homepage—Health, United States,” (2024), accessed October 15, 2024, https://www.cdc.gov/nchs/hus/index.htm.

[5] National Cancer Institute , “State Cancer Profiles>Quick Profiles,” (n.d.), accessed October 9, 2024, https://statecancerprofiles.cancer.gov/quick‐profiles/index.php?statename=southcarolina.

[6] American Cancer Society , “Colorectal Cancer Facts and Figures 2023–2025,” (2022).

[7] T. Wilkins , D. McMechan , and A. Talukder , “Colorectal Cancer Screening and Prevention,” American Family Physician 97, no. 10 (2018): 658–665. 29763272

[8] J. K. Triantafillidis , C. Vagianos , A. Gikas , M. Korontzi , and A. Papalois , “Screening for Colorectal Cancer: The Role of the Primary Care Physician,” European Journal of Gastroenterology & Hepatology 29, no. 1 (2017): e1–e7, https://journals.lww.com/eurojgh/fulltext/2017/01000/screening_for_colorectal_cancer__the_role_of_the.1.aspx. 27676092 10.1097/MEG.0000000000000759 PMC5134820

[9] US Preventive Services Task Force , “Screening for Colorectal Cancer: US Preventive Services Task Force Recommendation Statement,” Journal of the American Medical Association 325, no. 19 (2021): 1965–1977, 10.1001/jama.2021.6238. 34003218

[10] S. H. Rim , D. A. Joseph , C. B. Steele , T. D. Thompson , and L. C. Seeff , “Colorectal Cancer Screening—United States, 2002, 2004, 2006, and 2008,” MMWR Supplement 60, no. 1 (2011): 42–46. 21430619

[11] R. G. S. Meester , C. A. Doubeni , A. G. Zauber , et al., “Public Health Impact of Achieving 80% Colorectal Cancer Screening Rates in the United States by 2018,” Cancer 121, no. 13 (2015): 2281–2285, 10.1002/cncr.29336. 25763558 PMC4567966

[12] J. L. Moss , C. N. Pinto , S. Srinivasan , K. A. Cronin , and R. T. Croyle , “Persistent Poverty and Cancer Mortality Rates: An Analysis of County‐Level Poverty Designations,” Cancer Epidemiology, Biomarkers & Prevention 29, no. 10 (2020): 1949–1954, 10.1158/1055-9965.EPI-20-0007. PMC7534551 32998949

[13] J. Zhao , X. Han , L. Nogueira , et al., “Health Insurance Status and Cancer Stage at Diagnosis and Survival in the United States,” CA: A Cancer Journal for Clinicians 72, no. 6 (2022): 542–560, 10.3322/caac.21732. 35829644

[14] S. J. Henley , R. N. Anderson , C. C. Thomas , G. M. Massetti , B. Peaker , and L. C. Richardson , “Invasive Cancer Incidence, 2004–2013, and Deaths, 2006–2015, in Nonmetropolitan and Metropolitan Counties—United States,” MMWR Surveillance Summaries 66, no. 14 (2017): 1–13, 10.15585/mmwr.ss6614a1. PMC5879727 28683054

[15] C. A. Doubeni , S. Weinmann , K. Adams , et al., “Screening Colonoscopy and Risk for Incident Late‐Stage Colorectal Cancer Diagnosis in Average‐Risk Adults: A Nested Case‐Control Study,” Annals of Internal Medicine 158, no. 5 Pt 1 (2013): 312–320, 10.7326/0003-4819-158-5-201303050-00003. 23460054 PMC3752391

[16] R. Nishihara , K. Wu , P. Lochhead , et al., “Long‐Term Colorectal‐Cancer Incidence and Mortality After Lower Endoscopy,” New England Journal of Medicine 369, no. 12 (2013): 1095–1105, 10.1056/NEJMoa1301969. 24047059 PMC3840160

[17] C. A. Doubeni , D. A. Corley , V. P. Quinn , et al., “Effectiveness of Screening Colonoscopy in Reducing the Risk of Death From Right and Left Colon Cancer: A Large Community‐Based Study,” Gut 67, no. 2 (2018): 291–298, 10.1136/gutjnl-2016-312712. 27733426 PMC5868294

[18] 80% in Every Community , “American Cancer Society National Colorectal Cancer Roundtable,” accessed October 9, 2024, https://nccrt.org/our‐impact/80‐in‐every‐community/.

[19] E. D. Paskett and F. R. Khuri , “Can We Achieve an 80% Screening Rate for Colorectal Cancer by 2018 in the United States?,” Cancer 121, no. 13 (2015): 2127–2128, 10.1002/cncr.29335. 25764526 PMC4567968

[20] “The Guide to Community Preventive Services (The Community Guide),” (2024), accessed October 9, 2024, https://www.thecommunityguide.org/index.html.

[21] P. A. Hannon , A. E. Maxwell , C. Escoffery , et al., “Colorectal Cancer Control Program Grantees' Use of Evidence‐Based Interventions,” American Journal of Preventive Medicine 45, no. 5 (2013): 644–648, 10.1016/j.amepre.2013.06.010. 24139779 PMC4618374

[22] P. A. Hannon , A. E. Maxwell , C. Escoffery , et al., “Adoption and Implementation of Evidence‐Based Colorectal Cancer Screening Interventions Among Cancer Control Program Grantees, 2009–2015,” Preventing Chronic Disease 16 (2019): E139, 10.5888/pcd16.180682. 31603404 PMC6795067

[23] A. E. Maxwell , A. DeGroff , S. D. Hohl , et al., “Evaluating Uptake of Evidence‐Based Interventions in 355 Clinics Partnering With the Colorectal Cancer Control Program, 2015–2018,” Preventing Chronic Disease 19 (2022): E26, 10.5888/pcd19.210258. 35588522 PMC9165474

[24] E. M. Dias , J. R. Padilla , P. M. Cuccaro , et al., “Barriers to and Facilitators of Implementing Colorectal Cancer Screening Evidence‐Based Interventions in Federally Qualified Health Centers: A Qualitative Study,” BMC Health Services Research 24, no. 1 (2024): 797, 10.1186/s12913-024-11163-0. 38987761 PMC11238502

[25] T. L. Malo , S. Y. Correa , A. A. Moore , et al., “Centralized Colorectal Cancer Screening Outreach and Patient Navigation for Vulnerable Populations in North Carolina: Study Protocol for the SCORE Randomized Controlled Trial,” Implementation Science Communications 2, no. 1 (2021): 113, 10.1186/s43058-021-00194-x. 34620250 PMC8499575

[26] M. C. O'Leary , K. Hassmiller Lich , D. S. Reuland , et al., “Optimizing Process Flow Diagrams to Guide Implementation of a Colorectal Cancer Screening Intervention in New Settings,” Cancer Causes & Control 34, no. 1 (2023): 89–98, 10.1007/s10552-023-01769-w. PMC10689519 37731072

[27] C. Rohweder , M. Wangen , M. Black , et al., “Understanding Quality Improvement Collaboratives Through an Implementation Science Lens,” Preventive Medicine 129 (2019): 105859, 10.1016/j.ypmed.2019.105859. PMC7138534 31655174

[28] T. Abelsson , A. K. Karlsson , H. Morténius , A. Baigi , and S. Bergman , “The Dilemma of the Split Between Theory and Reality as Experienced by Primary Healthcare Professionals: A Mixed Methods Study of Evidence‐Based Practice in a Primary Care Context,” BMC Primary Care 25, no. 1 (2024): 13, 10.1186/s12875-023-02237-9. 38178021 PMC10768255

[29] N. Alsadaan and O. M. E. Ramadan , “Barriers and Facilitators in Implementing Evidence‐Based Practice: A Parallel Cross‐Sectional Mixed Methods Study Among Nursing Administrators,” BMC Nursing 24, no. 1 (2025): 403, 10.1186/s12912-025-03059-z. 40211261 PMC11987419

[30] Uniform Data System (UDS) Training and Technical Assistance|Bureau of Primary Health Care, accessed April 16, 2025, https://bphc.hrsa.gov/data‐reporting/uds‐training‐and‐technical‐assistance.

[31] National Committee for Quality Assurance , “HEDIS,” (2024), accessed December 3, 2024, https://www.ncqa.org/hedis/.

[32] K. P. Sharma , A. DeGroff , L. Scott , S. Shrestha , S. Melillo , and S. A. Sabatino , “Correlates of Colorectal Cancer Screening Rates in Primary Care Clinics Serving Low Income, Medically Underserved Populations,” Preventive Medicine 126 (2019): 105774, 10.1016/j.ypmed.2019.105774. 31319118 PMC6904949

[33] D. N. Owusu , E. A. Mensah , S. Mamudu , B. Brooks , and D. Shoham , “Rural–Urban Disparities in Colorectal Cancer Screening in United States: Blinder‐Oaxaca Decomposition Analysis of BRFSS Data,” Cancer Causes & Control 36, no. 12 (2025): 1911–1917, 10.1007/s10552-025-02071-7. 40971095

[34] J. M. Eberth , A. Thibault , R. Caldwell , et al., “A Statewide Program Providing Colorectal Cancer Screening to the Uninsured of South Carolina,” Cancer 124, no. 9 (2018): 1912–1920, 10.1002/cncr.31250. 29415338 PMC5910186

[35] B. J. Weiner , “A Theory of Organizational Readiness for Change,” Implementation Science 4 (2009): 67, 10.1186/1748-5908-4-67. 19840381 PMC2770024

[36] Azara Healthcare , “Azara Solutions,” accessed October 24, 2024, https://www.azarahealthcare.com/solutions.

[37] “Lucidchart|Diagramming Powered by Intelligence,” accessed October 24, 2024, https://www.lucidchart.com/pages/landing?utm_source=google&utm_medium=cpc&utm_campaign=_chart_en_us_mixed_search_brand_exact_&km_CPC_CampaignId=1457964857&km_CPC_AdGroupID=57044764032&km_CPC_Keyword=lucidchart&km_CPC_MatchType=e&km_CPC_ExtensionID=&km_CPC_Network=g&km_CPC_AdPosition=&km_CPC_Creative=354596046394&km_CPC_TargetID=kwd‐33511936169&km_CPC_Country=9198699&km_CPC_Device=c&km_CPC_placement=&km_CPC_target=&gad_source=1&gbraid=0AAAAADLdSjAZyI_fEWlaNCfULIQ0y41Ah&gclid=Cj0KCQjw4Oe4BhCcARIsADQ0cskfibCWCQOvzP4n4l1pOGynaZQJCXX9jvtv3AcXoMJOnwHs25CiEQAaAiT5EALw_wcB.

[38] R. Scoville and K. Little , “Comparing Lean and Quality Improvement|Institute for Healthcare Improvement,” accessed October 9, 2024, https://www.ihi.org/resources/white‐papers/comparing‐lean‐and‐quality‐improvement.

[39] S. Malone , K. Prewitt , R. Hackett , et al., “The Clinical Sustainability Assessment Tool: Measuring Organizational Capacity to Promote Sustainability in Healthcare,” Implementation Science Communications 2, no. 1 (2021): 77, 10.1186/s43058-021-00181-2. 34274004 PMC8285819

[40] “South Carolina Health Center Program Uniform Data System (UDS) Data,” accessed December 10, 2025, https://data.hrsa.gov/topics/healthcenters/uds/overview/state/SC.

